# Mechanistic Modeling of Placental Drug Transfer in Humans: How Do Differences in Maternal/Fetal Fraction of Unbound Drug and Placental Influx/Efflux Transfer Rates Affect Fetal Pharmacokinetics?

**DOI:** 10.3389/fped.2021.723006

**Published:** 2021-10-18

**Authors:** Xiaomei I. Liu, Dionna J. Green, John N. van den Anker, Natella Y. Rakhmanina, Homa K. Ahmadzia, Jeremiah D. Momper, Kyunghun Park, Gilbert J. Burckart, André Dallmann

**Affiliations:** ^1^Division of Clinical Pharmacology, Children's National Hospital, Washington, DC, United States; ^2^Office of Pediatric Therapeutics, Office of the Commissioner, US Food and Drug Administration, Silver Spring, MD, United States; ^3^Division of Infectious Diseases, Children's National Hospital, Washington, DC, United States; ^4^Technical Strategies and Innovation, Elizabeth Glaser Pediatric AIDS Foundation, Washington, DC, United States; ^5^Division of Maternal-Fetal Medicine, Department of Obstetrics and Gynecology, School of Medicine and Health Sciences, The George Washington University, Washington, DC, United States; ^6^Skaggs School of Pharmacy and Pharmaceutical Sciences, University of California, San Diego, La Jolla, CA, United States; ^7^Office of Clinical Pharmacology, US Food and Drug Administration, Silver Spring, MD, United States; ^8^Pharmacometrics/Modeling and Simulation, Research and Development, Pharmaceuticals, Bayer AG, Leverkusen, Germany

**Keywords:** physiologically based pharmacokinetics (PBPK), placental drug transfer, maternal-fetal, pregnancy, mechanistic modeling

## Abstract

**Background:** While physiologically based pharmacokinetic (PBPK) models generally predict pharmacokinetics in pregnant women successfully, the confidence in predicting fetal pharmacokinetics is limited because many parameters affecting placental drug transfer have not been mechanistically accounted for.

**Objectives:** The objectives of this study were to implement different maternal and fetal unbound drug fractions in a PBPK framework; to predict fetal pharmacokinetics of eight drugs in the third trimester; and to quantitatively investigate how alterations in various model parameters affect predicted fetal pharmacokinetics.

**Methods:** The ordinary differential equations of previously developed pregnancy PBPK models for eight drugs (acyclovir, cefuroxime, diazepam, dolutegravir, emtricitabine, metronidazole, ondansetron, and raltegravir) were amended to account for different unbound drug fractions in mother and fetus. Local sensitivity analyses were conducted for various parameters relevant to placental drug transfer, including influx/efflux transfer clearances across the apical and basolateral membrane of the trophoblasts.

**Results:** For the highly-protein bound drugs diazepam, dolutegravir and ondansetron, the lower fraction unbound in the fetus vs. mother affected predicted pharmacokinetics in the umbilical vein by ≥10%. Metronidazole displayed blood flow-limited distribution across the placenta. For all drugs, umbilical vein concentrations were highly sensitive to changes in the apical influx/efflux transfer clearance ratio. Additionally, transfer clearance across the basolateral membrane was a critical parameter for cefuroxime and ondansetron.

**Conclusion:** In healthy pregnancies, differential protein binding characteristics in mother and fetus give rise to minor differences in maternal-fetal drug exposure. Further studies are needed to differentiate passive and active transfer processes across the apical and basolateral trophoblast membrane.

## Introduction

Although drug use in pregnant women is frequent and increasing (1, 2), little is known about the different factors modulating placental transfer and fetal drug exposure. This knowledge is particularly important in an era where multiple approaches to therapy for the fetus are being considered (3, 4). As clinical studies involving pregnant women are difficult to conduct due to various considerations (5), other approaches are needed as alternative or complementary tools to elucidate maternal-fetal pharmacology. Among these tools, physiologically based pharmacokinetic (PBPK) modeling holds potential to improve the conceptual and quantitative understanding of maternal-fetal pharmacokinetics (6, 7). PBPK models integrate compound-specific properties (e.g., lipophilicity, molecular weight) and physiological and biological characteristics (e.g., organ volumes and blood flow rates) in a mechanistic framework (8). Whole-body PBPK models include multiple compartments which represent different organs and tissues that are arranged in a parallel circuit mimicking the blood flow in the circulatory system (9).

In recent years, numerous PBPK models for pregnant women have been developed and successfully evaluated with clinical data (10). Many of these models also described transfer of xenobiotics across the placenta and fetal pharmacokinetics (11). While much progress has been made in developing maternal-fetal PBPK models, many of these models lack a fully mechanistic description of the xenobiotic's placental transfer and partitioning between the maternal and fetal compartments. For example, differences in protein binding in maternal and fetal blood plasma have rarely been considered mechanistically. Yet, an altered fraction unbound of the drug in the fetal circulation might give rise to differences in drug exposure at steady-state, especially if the drug crosses the placenta exclusively *via* passive diffusion (12). Additionally, different influx and efflux transfer rates across the apical membrane of the trophoblast could be indicative of the presence of uptake or influx transporters (11, 13).

Hence, using a generic PBPK framework that can be extended to other drugs, the aims of this study were to (i) implement the unbound fraction of a drug in fetal model compartments; (ii) implement scaling factors for transplacental diffusion clearance that allow different influx and efflux transfer rates across the apical membrane of the trophoblasts; (iii) predict and evaluate maternal and fetal pharmacokinetics of a variety of drugs in the late third trimester with differential protein binding characteristics in the maternal and fetal organism when equal or different influx/efflux rates across the placenta are assumed; and (iv) quantify the effect of variations in maternal/fetal plasma protein binding, maternal blood flow rate to the placenta and placental influx/efflux rates on the predicted fetal pharmacokinetics through sensitivity analysis.

## Methods

### Software

PBPK models were built with PK-Sim® and MoBi® which are available as open source tools through the Open Systems Pharmacology (OSP) software, version 9.1, *via* GitHub (https://github.com/Open-Systems-Pharmacology) (14). The updated model files described herein will be also uploaded there. The software R, version 3.6.3 (R Foundation for Statistical Computing, http://www.r-project.org) was used for graphics creation. Clinical data were digitized from published figures using WebPlotDigitizer, version 4.4 (https://automeris.io/WebPlotDigitizer/).

### General Workflow

In previous studies, pregnancy PBPK models were built with the OSP software for the compounds acyclovir (15), cefuroxime (16), diazepam (17), dolutegravir (18), emtricitabine (15), metronidazole (17), ondansetron (17), and raltegravir (18). These models were successfully evaluated in non-pregnant adults, translated to pregnancy and the predicted maternal pharmacokinetics (16, 17) or predicted maternal and fetal pharmacokinetics at delivery (15, 18) were evaluated with clinical data. All models are freely available on OSP GitHub (https://github.com/Open-Systems-Pharmacology/Pregnancy-Models).

In this study, these models were used for further analyzing placental drug transfer. The development of additional non-pregnant PBPK models for other drugs and their extrapolation to and validation for pregnancy was beyond the scope of this study that focused exclusively on models that were already validated for pregnant women. Here, these models were updated by implementing the drug's fraction unbound in all fetal compartments as described in detail below. In contrast to previous studies (15, 18) the placental partition coefficients between maternal blood plasma and fetal intracellular space were predicted from the drug's physicochemical properties and the placental tissue composition. Additionally, the transfer clearance across the apical trophoblast membrane was estimated from *in vitro* permeability measures as described below. Apart from these changes, no other model adjustments were made. Pregnancy-induced changes in relevant anatomical and physiological model parameter values, including clearance values, can be found in previous publications (15–18). After these structural model updates, maternal and fetal pharmacokinetics were predicted using different values for the maternal and fetal unbound drug fraction. Transfer rates across the placenta were initially kept equal in both directions (symmetrical transfer). Thereafter, local sensitivity analyses were conducted by varying the maternal blood flow to the placenta and the influx and efflux rates across the placental membrane.

### Estimation of Fetal Fraction Unbound

Each drug's fraction unbound in fetal blood plasma (*f*_*u*_*fetus*_) was estimated using the following equation that has been evaluated for various populations, including infants (19) and pregnant women (16):


(1) $$\begin{array}{l} f_{u\text{\_}fetus}=1/\left(1+\frac{1-f_{u\text{\_}nonpreg}}{C_{prot\text{\_}nonpreg\;}\times f_{u\text{\_}nonpreg\;}}\times C_{prot\text{\_}fetus\;}\right) \end{array}$$

Here, *f*_*u*_*nonpreg*_ is the fraction unbound of non-pregnant adults in plasma; *C*_*prot*_*nonpreg*_ is the concentration of binding proteins in the blood plasma in non-pregnant adults; *C*_*prot*_*fetus*_ is the concentration of binding proteins in fetal blood plasma. Values for *C*_*prot*_*fetus*_ were taken from a previously published meta-analysis (20). Implicit assumptions of this equation are that the number of adult and fetal protein binding sites and the drug's affinity to adult and fetal plasma proteins are the same and that the drug exclusively binds to one plasma protein only. Table 1 lists for each drug the observed fraction unbound in non-pregnant subjects (*f*_*u*_*nonpreg*_) and the estimated maternal and fetal fraction unbound implemented in the PBPK model.

**Table 1:** Overview of the observed fraction unbound in non-pregnant subjects and the estimated fraction unbound in mother and fetus.

**Drug**	**Fraction unbound in non-pregnant subjects**	**Maternal fraction unbound**	**Fetal fraction unbound**
Acyclovir	0.85 (15)	0.88	0.86
Cefuroxime	0.67 (16)	0.73	0.68
Diazepam	0.020 (17)	0.027	0.021
Dolutegravir	0.0070 (18)	0.0088	0.0080
Emtricitabine	0.96 (15)	0.97	0.96
Metronidazole	0.89 (17)	0.92	0.89
Ondansetron	0.27 (17)	0.33	0.28
Raltegravir	0.17 (18)	0.24	0.23

### Structural Implementation of the Fetal Fraction Unbound in the Model

**Figure 1:**
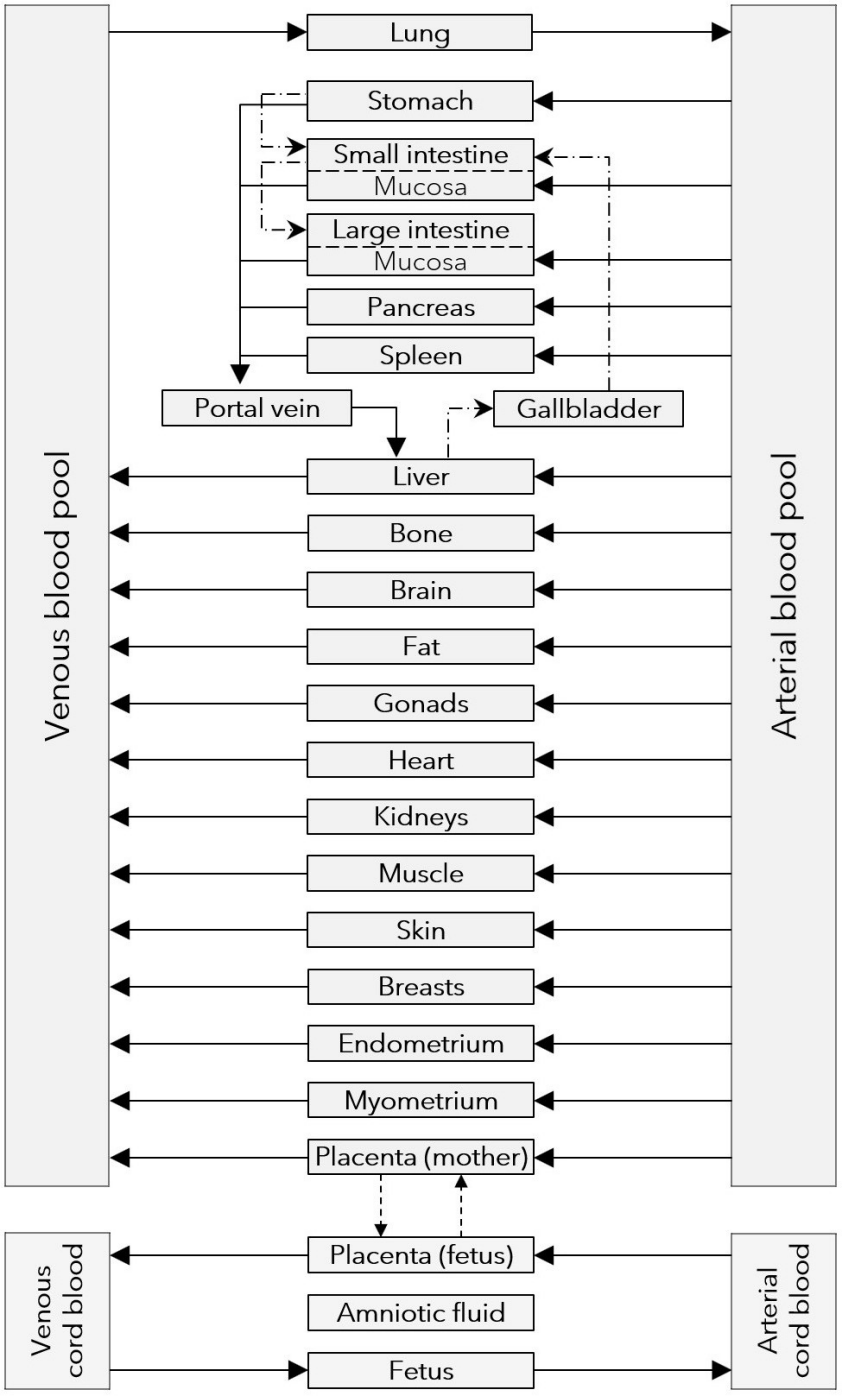
Structure of the pregnancy PBPK models. Gray boxes represent compartments of the PBPK model; solid arrows denote drug transport *via* the organ blood flow; dashed arrows denote drug transport *via* passive diffusion; dash dotted lines denote drug transport *via* gastrointestinal motility or the biliary excretion route. PBPK, physiologically based pharmacokinetic.

The structure of the pregnancy PBPK model is schematically shown in Figure 1 and has been described in detail previously (16). Briefly, the fetal sub-structure of the pregnancy PBPK model consists of five compartments representing the fetal part of the placenta, the fetus, the amniotic fluid volume (which is not connected to other compartments) and the arterial and venous blood pools of the umbilical cord. Organ compartments are further sub-divided in the blood cells (*bc*), plasma (*pls*), interstitial (*int*), and intracellular compartment (*cell*).

Here, the ordinary differential equations (ODE) system of the fetal sub-structure was refined in MoBi® to account for fetal-specific protein binding. In MoBi® the ODEs are first defined for intercompartmental exchange processes in the passive transports building block; during set-up of a simulation, the ODEs are then generated for each compartment. In the following, the ODEs are first introduced for each intercompartmental exchange transport and then defined for the compartments.

Specifically, the ODEs in the fetal compartments describing drug exchange between plasma and blood cells (Equation 2), plasma and interstitial space (Equaton 3), and interstitial and intracellular space (Equation 4) were adjusted in the spatial structure building block section of MoBi® as described below. Note that Equations (2–4) only refer to the passive, gradient-driven drug exchange between the two sub-compartments.


(2) $$\begin{array}{ll} \frac{dN_{bc}}{dt} & =SA_{bc}\times P_{pls,bc}\times f_{u}\times \left(C_{pls}-\frac{C_{bc}}{K_{bc}}\right) \end{array}$$

(3) $$\begin{array}{ll} \frac{dN_{int}^{int↔pls}}{dt} & =P_{end}\times SA_{end}\times f_{u}\times \left(C_{pls}-\frac{C_{int}}{K_{int,pls}}\right) \end{array}$$

(4) $$\begin{array}{l} \frac{dN_{cell}}{dt}=P_{int,cell}\times SA_{int,cell}\\ \;\times \left(K_{water,int}\times C_{int}-K_{water,cell}\times C_{cell}\right) \end{array}$$

Here, *N*_*bc*_ denotes molar drug amount in blood cells (μmol), $N_{int}^{int↔pls}$ denotes molar drug amount in the interstitial compartment when only drug exchange between plasma and interstitial is considered (μmol); *N*_*cell*_ denotes molar drug amount in the intracellular compartment (μmol); *C*_*bc*_, *C*_*cell*_, *C*_*int*_ and *C*_*pls*_ denote the molar drug concentration in blood cells, intracellular space, interstitial space, and plasma, respectively (μmol/L); *f*_*u*_ the drug's fraction unbound in maternal blood plasma (which was originally assumed to be equal with the fraction unbound in fetal blood plasma); *K*_*bc*_, *K*_*int,pls*_, *K*_*water,cell*_ and *K*_*water,int*_ the partition coefficient between blood cells and plasma, interstitial and plasma, water and intracellular space and between water and interstitial space, respectively; *N* the molar drug amount (μmol); *P*_*end*_, *P*_*int,cell*_ and *P*_*pls,bc*_ the drug's permeability through the endothelial, cellular, and blood cell membrane, respectively (assuming symmetrical transfer, i.e., equal permeability for both directions) (cm/min); and *SA*_*bc*_, *SA*_*end*_, and *SA*_*int,cell*_ the total surface area of the endothelial, cellular and blood cell membrane, respectively (cm^2^). The parameterization can be found elsewhere (16).

To account for the fetus-specific fraction of unbound drug in the model, Equations (2–4) were amended as described in the following. In all equations, *f*_*u*_ (the maternal fraction unbound) was substituted with *f*_*u*_*fetus*_ (the fetal fraction unbound) calculated from Equation (1).

Assuming that *K*_*bc*_ in Equation (2) is the same for the maternal and fetal organism and substituting *f*_*u*_ with *f*_*u*_*fetus*_ yields Equation (5).


(5) $$\begin{array}{l} \frac{dN_{bc}}{dt}=SA_{bc}\times P_{pls,bc}\times f_{u\text{\_}fetus}\times \left(C_{pls}-\frac{C_{bc}}{K_{bc}}\right) \end{array}$$

In Equation (3), *K*_*int,pls*_ was calculated according to the equation reported by Schmitt (21):


(6) $$\begin{array}{l} K_{int,pls}=\left(f_{water\text{\_}int}+\frac{f_{prot\text{\_}int}}{f_{prot\text{\_}pls}}\times \left(\frac{1}{f_{u\text{\_}fetus}}-f_{water\text{\_}pls}\right)\right)\times f_{u\text{\_}fetus} \end{array}$$

where *f*_*water*_*pls*_ and *f*_*water*_*int*_ denote the fractional volume content of water in plasma and interstitial space, respectively; and *f*_*prot*_*pls*_ and *f*_*prot*_*int*_ denote the fractional volume content of proteins in plasma and interstitial space, respectively. Of note, *f*_*water*_*pls*_, *f*_*water*_*int*_ and the ratio $\frac{f_{prot\text{\_}int}}{f_{prot\text{\_}pls}}$ were assumed to be the same as in the adult organism, namely 0.926 (22), 0.935 (22), and 0.37 (23), respectively. Inserting Equation (6) into Equation (3) yields Equation (7) which was used in the updated maternal-fetal PBPK model.


(7) $$\begin{array}{l} \frac{dN_{int}^{int↔pls}}{dt}=P_{end}\times SA_{end}\times f_{u\text{\_}fetus}\\ \times \left(C_{pls}-\frac{C_{int}}{\left(f_{water\text{\_}int}+\frac{f_{prot\text{\_}int}}{f_{prot\text{\_}pls}}\times \left(\frac{1}{f_{u\text{\_}fetus}}-f_{water\text{\_}pls}\right)\right)\times f_{u\text{\_}fetus}}\right) \end{array}$$

To refine Equation (4), *K*_*water,int*_ and *K*_*water,cell*_ were expressed as:


(8) $$\begin{array}{l} K_{water,int}=\frac{f_{u\text{\_}fetus}}{K_{int,pls}} \end{array}$$

(9) $$\begin{array}{l} K_{water,cell}=\frac{f_{u\text{\_}fetus}}{K_{cell,pls}} \end{array}$$

*K*_*int,pls*_ is calculated according to Equation (6), while several equations were reported for predicting *K*_*cell,pls*_, namely the PK-Sim Standard equation (24) and the equations proposed by Schmitt et al. (21), Rodgers et al. (25, 26), and Poulin et al. (27, 28). These equations—subsequently referred to as PK-Sim Standard, Schmitt, Rodgers and Rowland, and Poulin and Theil model—are implemented per default in the OSP software and use the global (i.e., maternal) fraction unbound which appears as a discrete multiplier in these equations. Hence, instead of manually changing the underlying equations, the default equations using the maternal fraction unbound were kept and Equation (9) were multiplied with the ratio of maternal to fetal fraction unbound as a correction factor ($\frac{f_{u}}{f_{u\text{\_}fetus}}$) so that the maternal fraction unbound cancels out and the fetal fraction unbound is included in the denominator:


(10) $$\begin{array}{l} K_{water,cell}=\frac{f_{u\text{\_}fetus}}{K_{cell,pls}}\times \frac{f_{u}}{f_{u\text{\_}fetus}}\; \end{array}$$

Finally, inserting Equations (6, 8, and 10) in Equation (4) yields Equation (11):


(11) $$\begin{array}{l} \frac{dN_{cell}}{dt}=P_{int,cell}\times SA_{int,cell}\\ \;\times \left(C_{int}\times \frac{1}{f_{water\text{\_}int}+\frac{f_{prot\text{\_}int}}{f_{prot\text{\_}pls}}\times \left(\frac{1}{f_{u\text{\_}fetus}}-f_{water\text{\_}pls}\right)}-C_{cell}\times \frac{f_{u}}{K_{cell,pls}}\right) \end{array}$$

Fetal-specific changes of parameters appearing in Equation 11 and input variables thereof, such as the volume fraction of water and lipids in each tissue compartment which are needed to calculate *K*_*cell,pls*_, were also accounted for. Quantitative data on these input variables was previously gathered from the literature (29). Equations (5, 7, and 11) were then used for all fetal compartments in the PBPK model.

Hence, the full equations for the rate of change of drug amount in the four compartments implemented in the updated maternal-fetal PBPK model were as follows:


(12) $$\begin{array}{l} d_{t}\left[\begin{array}{c} N_{bc}\\ \begin{array}{c} N_{pls}\\ \begin{array}{c} N_{int}\\ N_{cell} \end{array} \end{array} \end{array}\right]=Q\left[\begin{array}{c} HCT\;\times \left(C_{bc}^{bp\text{\_}inflow}-C_{bc}\right)\\ \begin{array}{c} \left(1-HCT\right)\times \left(C_{pls}^{bp\text{\_}inflow}-C_{pls}\right)\\ \begin{array}{c} 0\\ 0 \end{array} \end{array} \end{array}\right]+E\left[\begin{array}{c} C_{bc}\\ \begin{array}{c} C_{pls}\\ \begin{array}{c} C_{int}\\ C_{cell} \end{array} \end{array} \end{array}\right] \end{array}$$

Here, *Q* denotes the blood flow of the compartment (L/min); *HCT* the hematocrit; $C_{bc}^{bp\text{\_}inflow}$ and $C_{bc}^{bp\text{\_}inflow}$ the molar drug concentration in the blood cells and plasma, respectively, of the blood pools that supplies the current compartment with blood (e.g., venous blood pool of the umbilical cord) (μmol/L) and *E* the passive drug exchange between the compartments driven by concentration gradients. Specifically, *E* was defined as the following 4 × 4 matrix:


(13) $$\begin{array}{l} E=f_{u\text{\_}fetus}\left[\begin{array}{llll} -P_{pls,bc}\times \frac{SA_{bc}}{K_{bc}} & P_{pls,bc}\times SA_{bc} & 0 & 0\\ P_{pls,bc}\times \frac{SA_{bc}}{K_{bc}} & -P_{pls,bc}\times SA_{bc}-P_{end}\times SA_{end} & P_{end}\times \frac{SA_{end}}{K_{int,pls}} & 0\\ 0 & P_{end}\times SA_{end} & \;-P_{end}\times \frac{SA_{end}}{K_{int,pls}}-P_{int,cell}\times \frac{SA_{int,cell}}{K_{int,pls}} & P_{int,cell}\times \frac{SA_{int,cell}\times f_{u}}{f_{u\text{\_}fetus}\times K_{cell,pls}}\\ 0 & 0 & P_{int,cell}\times \frac{SA_{int,cell}}{K_{int,pls}} & -P_{int,cell}\times \frac{SA_{int,cell}\times f_{u}}{f_{u\text{\_}fetus}\times K_{cell,pls}} \end{array}\right] \end{array}$$

### Structural Implementation of Maternal-Fetal Drug Transfer in the Model

**Figure 2:**
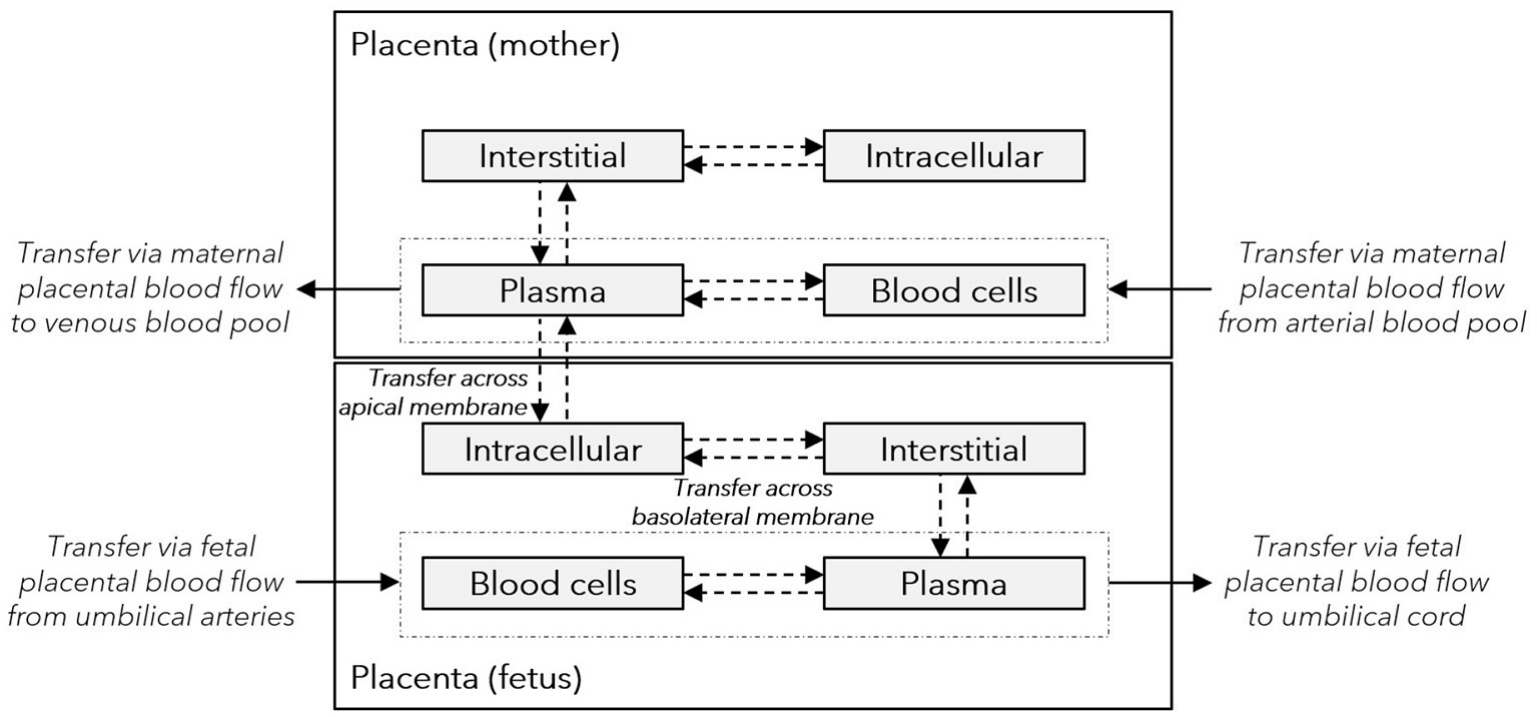
Sub-structure of the placenta barrier in the pregnancy PBPK models. Gray boxes represent sub-compartments of the placenta structure implemented in the pregnancy PBPK model; dash-dotted boxes represent the vascular space; solid arrows denote drug transport *via* the organ blood flow; and dashed arrows denote drug transport *via* passive diffusion. The intracellular compartment in the fetal placenta represents the (syncytio)trophoblasts with the apical membrane facing toward the maternal plasma compartment and the basolateral membrane toward the fetal interstitial compartment. PBPK, physiologically based pharmacokinetic.

The sub-structure of the placenta barrier embedded in the pregnancy PBPK model is schematically illustrated in Figure 2. In this structure, maternal-fetal drug transfer occurs *via* the apical membrane of the trophoblast (represented in the model structure by the fetal intracellular compartment of the placenta). The ODE to describe maternal-fetal drug transfer was extended by adding scaling factors (*f*_*in*_ and *f*_*out*_):


(14) $$\begin{array}{l} \frac{dN_{cell\text{\_}F}^{cell\text{\_}F↔pls\text{\_}M}}{dt}=f_{in}\times P_{pls,cell\text{\_}T}\times SA_{villi}\times f_{u}\times C_{pls\text{\_}M}-f_{out}\\ \;\times P_{pls,cell\text{\_}T}\times SA_{villi}\times f_{u}\times \frac{C_{cell\text{\_}F}}{K_{cell,pls}} \end{array}$$

In this equation, $N_{cell\text{\_}F}^{cell\text{\_}F↔pls\text{\_}M}$ is the molar drug amount in the fetal intracellular space of the placenta (trophoblasts) when only the maternal-fetal drug exchange process is considered (μmol); *f*_*in*_ and *f*_*out*_ are influx and efflux scaling factors, respectively; *P*_*pls,cell*_*T*_ is the permeability across the apical membrane of the trophoblasts (cm/min); *SA*_*villi*_ is the surface area of the fetal villi (cm^2^); *C*_*pls*_*M*_ and *C*_*cell*_*F*_ is the molar drug concentration in the maternal blood plasma of the placenta and fetal intracellular sub-space of the placenta, respectively (μmol/L); and *K*_*cell,pls*_ is the placental partition coefficient between the fetal intracellular and the maternal blood plasma sub-space.

For *K*_*cell,pls*_, the same calculation method is used as for the other compartments in the PBPK model, i.e., the PK-Sim Standard, Schmitt, Rodgers and Rowland, or Poulin and Theil method. Information on the placenta tissue composition has been reported previously (29). The product of *P*_*pls,cell*_ × *SA*_*villi*_, also termed apical transfer clearance, was calculated according to a previously published *in vitro-to-in vivo* extrapolation approach (30). This approach uses midazolam as an *in vivo* calibrator and scales the passive diffusion clearance of another drug from its apparent permeability across epithelial cell lines (e.g., Caco-2 cells). An exception was emtricitabine; since no apparent *in vitro* permeability value could be found in the literature, the product of *P*_*pls,cell*_ × *SA*_*villi*_ was set to a previously reported value (15) estimated based on the *ex vivo* cotyledon perfusion assay (31). Table 2 lists for each drug the method for predicting the tissue-to-plasma partition coefficients in the PBPK model together with the predicted value for *K*_*cell,pls*_ between the fetal intracellular and the maternal blood plasma compartment as well as the values for the apparent permeability in Caco-2 cells and the resulting apical transfer clearance. It should be stressed that this clearance refers to the drug transfer across the apical membrane of the fetal trophoblasts. Of note, the selection of a method for predicting the partition coefficients of a given drug (as listed in Table 2) was done during development of the non-pregnant, adult PBPK model. During this process, several partition coefficient methods were tested. The method with the best simulation result (i.e., lowest squared error) was chosen and subsequently used in the maternal-fetal PBPK model [further information on the development of the non-pregnant, adult PBPK models can be found elsewhere (15–18)].

**Table 2:** Overview of the methods for predicting the tissue-to-plasma partition coefficients of each drug in the PBPK model and placental transfer model parameters.

**Drug**	**Method for predicting tissue-to-plasma partition coefficients in the PBPK model**	**Placental partition coefficient**	**Caco-2 cell permeability (cm/s)**	**Apical transfer clearance (L/min)**
Acyclovir	PK-Sim Standard (24)	0.74	0.3 × 10^−6^ (32)	0.059
Cefuroxime	Schmitt (21)	0.61	1.2 × 10^−6^ (33)	0.20
Diazepam	PK-Sim Standard (24)	0.079	8.9 × 10^−5^ (34)	15.1
Dolutegravir	Rodgers and Rowland (25, 26)	0.16	2.5 × 10^−6^ (35)	0.43
Emtricitabine	Rodgers and Rowland (25, 26)	0.83	NA	0.019 (31)
Metronidazole	Rodgers and Rowland (25, 26)	0.80	5.7 × 10^−5^ (36)	9.76
Ondansetron	Poulin and Theil (27, 28)	0.41	1.8 × 10^−5^ (37)	3.11
Raltegravir	Rodgers and Rowland (25, 26)	0.28	7.3 × 10^−6^ (38)	1.24

*NA, not available; PBPK, physiologically based pharmacokinetic*.

Note that in the original model, *f*_*in*_ and *f*_*out*_ in Equation (14) have a value of 1 and that for values <1 the apical transfer clearance is reduced, whereas for values >1 the apical transfer clearance is increased. In addition, similar scaling factors were introduced in the equation describing transfer across the basolateral membrane of the trophoblast (Equation 4), i.e., from the fetal intracellular compartment to the interstitial compartment in the placenta (see Figure 2).

### Clinical Data

Clinical data for the investigated drugs herein were taken from the literature (18, 39–47) and are listed for each drug in Table 3. Blood samples were obtained from maternal peripheral venous blood and umbilical vein blood plasma at delivery. The timing of blood sampling relative to dose administration was highly heterogeneous due to the random nature in the time of delivery. Hence, for some drugs, e.g., acyclovir, cefuroxime and diazepam, few or no clinical data were available for the early absorption/distribution phase, whereas for others, e.g., ondansetron and metronidazole, few or no observed data were available in the elimination phase at delivery. Of note, clinical studies for diazepam investigated pharmacokinetics after doses of 5 mg (46) and 10 mg (45, 47) and studies for cefuroxime doses of 1,500 mg (42) and 750 mg (41). Here, the reported concentrations of diazepam and cefuroxime were normalized to the 10 and 750 mg dose, respectively, assuming linear pharmacokinetics.

**Table 3:** Characteristics of the clinical studies used for model evaluation.

**Drug**	**Posology**	**Gestational age (weeks)**	**Maternal concentration values (*n*)**	**Fetal concentration values (*n*)**	**References**
Acyclovir	400 mg TID, oral	39.9 [37–41]^a^	9	19	(39)
Cefuroxime	750 mg single dose, IV	41 [37–42]^b^	14	8	(41)
Cefuroxime	1,500 mg single dose, IV	32 [28–35]^b^	22	7	(42)
Diazepam	10 mg single dose, IV	38 [37–40]^b^	16	16	(45)
Diazepam	5 mg single dose, IV	38–40^c^	5	5	(46)
Diazepam	10 mg single dose, IV	NA^d^	6	6	(47)
Dolutegravir	50 mg QD, oral	38 [35–42]^b^	20	20	(18)
Emtricitabine	400 mg single dose, oral	39 [33–42]^b^	166	37	(40)
Metronidazole	500 mg single dose, IV	NA^d^	21	12	(44)
Ondansetron	4 mg single dose, IV	39.1 [36.4–40.4]^a^	46	9	(43)
Raltegravir	400 mg BID, oral	38 [36–40]^b^	20	20	(18)

a *Expressed as arithmetic mean (range)*.

b *Expressed as median (range)*.

c *Expressed as range; median not reported*.

d *Gestational age at delivery not reported; in the model a gestational age of 40 weeks was assumed*.

*BID, twice daily; IV, intravenous; NA, not available; PBPK, physiologically based pharmacokinetic; QD, once daily; TID, three times daily*.

### Evaluation of Predictive Model Performance

Pharmacokinetics was predicted at delivery in a virtual population of 500 pregnant women. The predictive model performance was assessed by visual comparison of predicted drug concentrations in the maternal blood plasma and the umbilical vein blood plasma at delivery with the clinical data described above and listed in Table 3. In addition to visual assessment of the predictive model performance, the mean prediction error (MPE) (%) and mean squared error (MSE) were calculated as follows:


(15) $$\begin{array}{l} MPE=\frac{100}{n}\sum \frac{C_{sim,i}-C_{obs,i}}{C_{obs,i}} \end{array}$$

(16) $$\begin{array}{l} MSE=\frac{1}{n}\sum {\left(C_{obs,i}-C_{sim,i}\right)}^{2} \end{array}$$

where *C*_*sim,i*_ and *C*_*obs,i*_ is the simulated and observed concentration at timepoint *i*, respectively; and *n* the total number of observed concentrations.

### Sensitivity Analysis

Local sensitivity analyses were conducted using the updated maternal-fetal PBPK models to assess quantitatively how changes in various model parameters propagate to the model output. For each drug, the blood flow rate to the maternal intervillous space in the placenta was varied by factors of 0.5 and 2. Additionally, the apical and basolateral transfer clearance was varied as follows. For the apical transfer clearance, the scaling factors *f*_*in*_ and *f*_*out*_ in Equation (14) were varied both together by factors ranging from 2 to 10 and separately from each other (i.e., affecting either influx or efflux transfer clearance) by factors ranging from 0.5 to 2. The basolateral transfer clearance was varied by multiplying the product of *P*_*int,cell*_ × *SA*_*int,cell*_ (i.e., the transfer clearance) in Equation (4) by factors ranging from 1.5 to 10. Either the apical or the basolateral transfer clearance was modified during sensitivity analysis but not both at the same time.

The effect of variations in these parameters were only tested using the predicted umbilical vein concentrations as model output; maternal concentration predictions were not included as model output as it was previously shown that large changes in various placental transfer parameters (namely in the permeability across the apical trophoblast membrane and partition coefficient) did not significantly impact maternal plasma concentrations (18, 48).

Additionally, the area under the concentration-time curve from time zero (or, in case of multiple dose studies, time of last dose administration) to the time of the last observed concentration (AUC_tlast_) was calculated from the observed data in maternal plasma and umbilical vein and compared with the predicted values.

## Results

**Figure 3:**
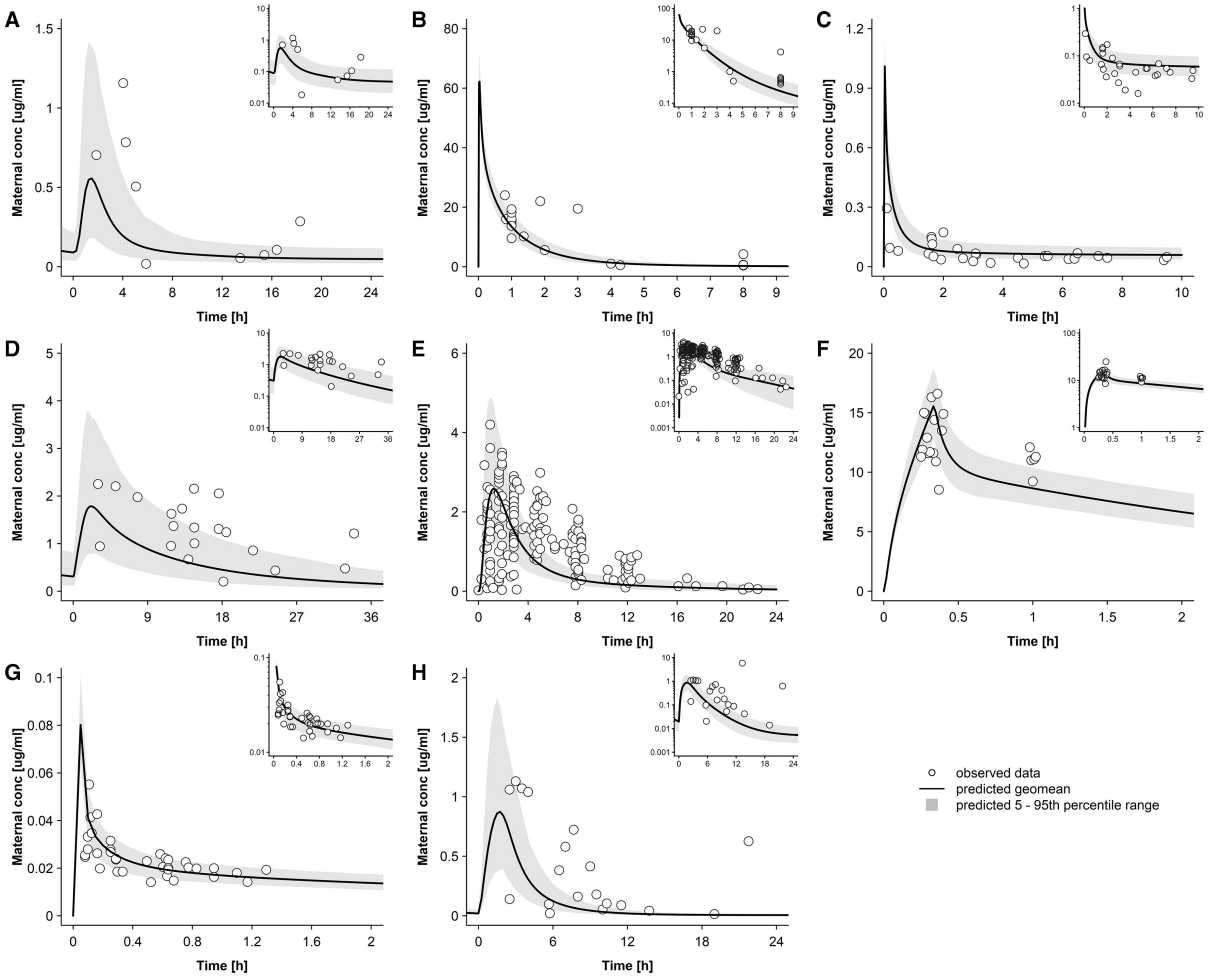
Maternal plasma concentration–time profiles observed and predicted at delivery. Concentration-time profiles in the maternal peripheral blood plasma for acyclovir **(A)**, cefuroxime **(B)**, diazepam **(C)**, dolutegravir **(D)**, emtricitabine **(E)**, metronidazole **(F)**, ondansetron **(G)**, and raltegravir **(H)** at delivery in the third trimester. Semi-log scaled figures are shown as inset plot in the top-right corner of each panel. The time refers to the time after drug administration. Circles represent individual observed data; the solid line represents the predicted geomean and the shaded area the predicted 5th−95th percentile range. Observed data for acyclovir, cefuroxime, diazepam, dolutegravir, emtricitabine, metronidazole, ondansetron, and raltegravir were taken from Liu et al. (18), Leung et al. (39), Hirt et al. (40), Philipson and Stiernstedt (41), De Leeuw et al. (42), Elkomy et al. (43), Visser and Hund (44), Moore and McBride (45), Ridd et al. (46), and Mandelli et al. (47), respectively. conc, concentration.

The observed and predicted maternal plasma concentration-time profiles are shown in Figure 3. While the predicted median concentration-time profiles generally captured the clinical data, the observed interindividual variability was underestimated by the models. Table 4 lists the MPEs for these predictions.

**Table 4:** Mean prediction errors and mean squared errors.

**Drug**	**Maternal plasma concentrations**		**Umbilical vein concentrations**	
	**Mean prediction error (%)**	**Mean squared error**	**Mean prediction error (%)**	**Mean squared error**
Acyclovir	16.9	0.17	15.7	0.12
Cefuroxime	−22.2	30.5	45.4	4.97
Diazepam	37.9	0.01	24.6	0.01
Dolutegravir	−37.2	0.68	−43.4	1.17
Emtricitabine	102.5	0.97	3.1	0.25
Metronidazole	0.9	13.5	−3.2	8.61
Ondansetron	4.4	7.0 × 10^−5^	−41.2	2.0 × 10^−5^
Raltegravir	−22.8	0.16	−23.3	0.51

**Figure 4:**
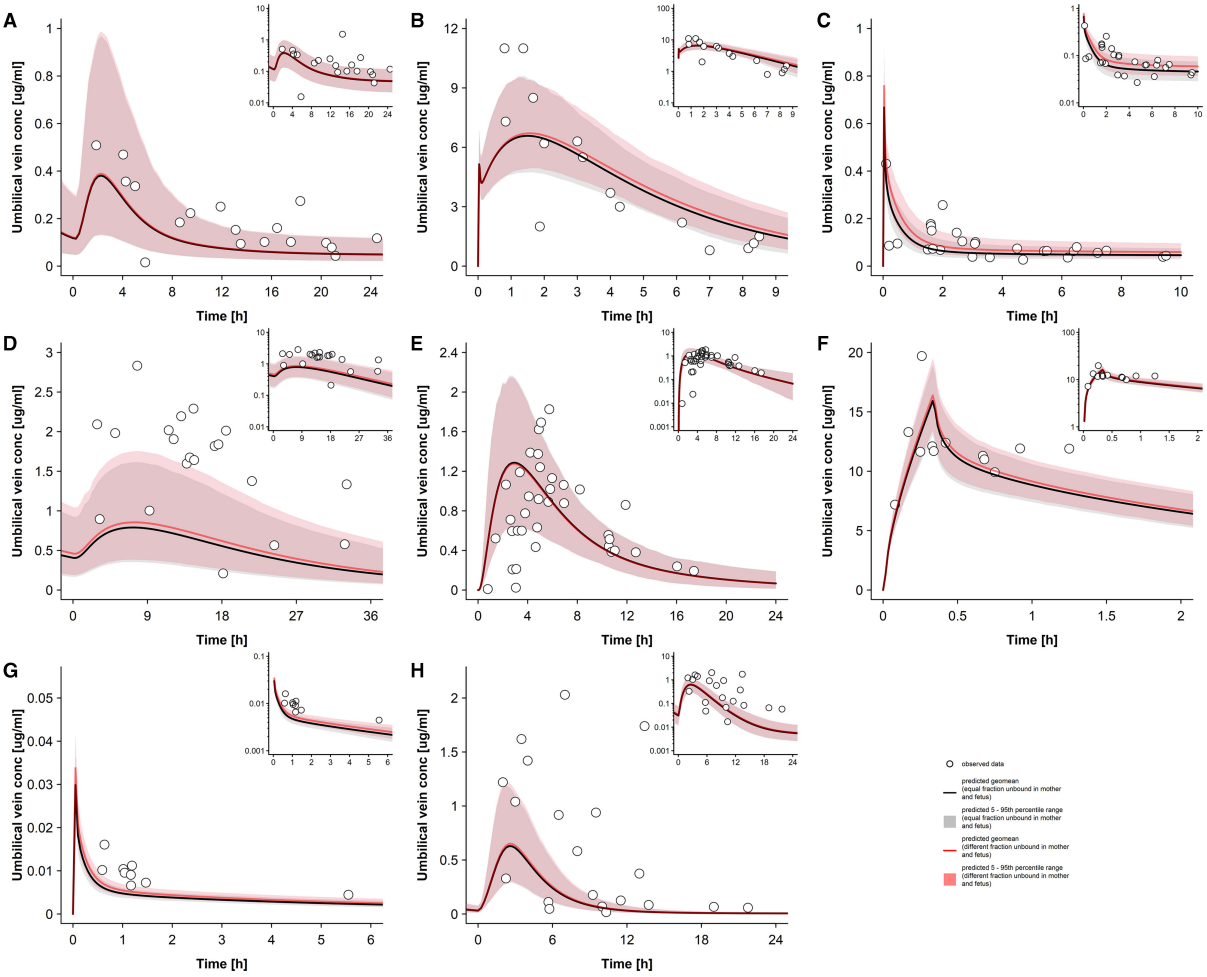
Umbilical vein plasma concentration–time profiles observed and predicted at delivery. Concentration-time profiles in the umbilical vein plasma for acyclovir **(A)**, cefuroxime **(B)**, diazepam **(C)**, dolutegravir **(D)**, emtricitabine **(E)**, metronidazole **(F)**, ondansetron **(G)**, and raltegravir **(H)** at delivery in the third trimester. Semi-log scaled figures are shown as inset plot in the top-right corner of each panel. The time refers to the time after drug administration. Circles represent individual observed data; the solid line represents the predicted geomean and the shaded area the predicted 5th−95th percentile range. Red shades denote to predictions with different maternal/fetal unbound drug fractions and black/gray colors predictions with equal maternal and fetal unbound drug fractions. Observed data for acyclovir, cefuroxime, diazepam, dolutegravir, emtricitabine, metronidazole, ondansetron, and raltegravir were taken from Liu et al. (18), Leung et al. (39), Hirt et al. (40), Philipson and Stiernstedt (41), De Leeuw et al. (42), Elkomy et al. (43), Visser and Hund (44), Moore and McBride (45), Ridd et al. (46), and Mandelli et al. (47), respectively. conc, concentration.

Figure 4 presents the observed and predicted plasma concentration-time profiles in the umbilical cord and Table 4 lists the MPEs and MSEs for these predictions. For some drugs, such as ondansetron and metronidazole, visual assessment of the fetal prediction results was only possible within a relatively narrow time interval after dose administration because of lacking clinical data at later time points. Therefore, the predicted elimination phase could not be evaluated. For other drugs, e.g., acyclovir, few clinical data were available in the early distribution phase (i.e., before reaching the peak concentration in the fetus). No consistent trend for under- or overestimation was evident across the different predicted pharmacokinetic profiles. While for some drugs, such as diazepam and ondansetron, the pharmacokinetic profiles were overall adequately captured, the clinical data were underestimated for other drugs, e.g., acyclovir and dolutegravir, or, in the case of emtricitabine, overestimated. Of note, the relatively high MPE for emtricitabine (102.5%, see Table 4) could predominantly be attributed to two observed plasma concentrations in the absorption phase that were substantially overestimated (specially these concentrations were 0.0095 and 0.024 μg/mL at 0.8 and 3 h, respectively). For cefuroxime the clinical data showed high variability and contained very limited information, so that an adequate assessment of the predicted umbilical cord concentration was difficult. Similar to the pharmacokinetics predicted in maternal plasma, the observed interindividual variability was generally underestimated by the models.

Note that Figure 4 also includes the prediction results that are obtained when setting the fetal fraction unbound value to that in the mother (i.e., equal fraction unbound values) to allow a visual assessment of the effect of plasma protein binding differences between the fetal and maternal circulatory system. For most drugs, differences in plasma protein binding between the mother and fetus translated into rather small differences in predicted umbilical cord concentrations. Table 5 lists the predicted AUC_tlast_ for the umbilical cord plasma concentrations obtained when assuming equal maternal/fetal unbound drug fractions and when considering differential maternal/fetal protein binding. As can be seen in this table, the effect of assuming a different fetal fraction unbound on AUC_tlast_ was below 5% for some drugs but exceeded 10% in the case of highly-protein bound drugs (diazepam, dolutegravir and ondansetron); raltegravir was an exception, though. Table 6 lists the observed and predicted AUC_tlast_ in maternal and umbilical vein plasma (when assuming differential maternal-fetal protein binding). In all but four cases, the observed AUC_tlast_ fell within the predicted 5th−95th percentile range.

**Table 5:** Predicted drug exposure in umbilical vein plasma with equal and different maternal-fetal protein binding.

**Drug**	**AUC_**tlast**_ predicted in umbilical vein plasma with equal maternal and fetal fraction unbound (μg h/mL)**	**AUC_**tlast**_ predicted in umbilical vein plasma with different maternal and fetal fraction unbound (μg h/mL)**	**Difference (%)**
Acyclovir	1.9	2.0	5.3
Cefuroxime	36.5	38.2	4.7
Diazepam	0.67	0.85	26.9
Dolutegravir	15.0	16.5	10.0
Emtricitabine	10.2	10.1	−0.98
Metronidazole	12.3	12.7	3.3
Ondansetron	0.024	0.028	16.7
Raltegravir	3.0	3.2	6.7

*AUC_tlast_, area under the concentration-time curve from time zero (or, in case of multiple dose studies, time of last dose administration) to the time of the last observed concentration*.

**Table 6:** Observed and predicted drug exposure in mother and fetus.

**Drug**	**AUC**_**tlast**_ **in maternal plasma (μg h/mL)**		**AUC**_**tlast**_ **in umbilical vein plasma (μg h/mL)**	
	**Observed**	**Predicted (geomean [*p*5; *p*95])**	**Observed**	**Predicted (geomean [*p*5; *p*95])**
Acyclovir	3.7	1.9 [0.66; 5.1]	5.3	2.0 [0.70; 5.1]
Cefuroxime	39.0	43.9 [32.8; 61.9]	33.3	38.2 [27.5; 54.4]
Diazepam	0.50	0.86 [0.55; 1.4]	0.67	0.85 [0.55; 1.4]
Dolutegravir	37.5	19.4 [8.2; 42.9]	37.8	16.5 [7.2; 35.1]
Emtricitabine	15.9	10.6 [5.0; 19.7]	10.7	10.1 [5.3; 16.9]
Metronidazole	7.0	5.1 [4.2; 6.2]	13.8	12.7 [10.4; 15.2]
Ondansetron	0.11	0.10 [0.074; 0.12]	0.030	0.028 [0.022; 0.036]
Raltegravir	5.9	2.9 [1.3; 6.3]	7.7	3.2 [2.4; 6.3]

*AUC_tlast_, area under the concentration-time curve from time zero (or, in case of multiple dose studies, time of last dose administration) to the time of the last observed concentration; p5, 5th percentile; p95, 95th percentile*.

**Figure 5:**
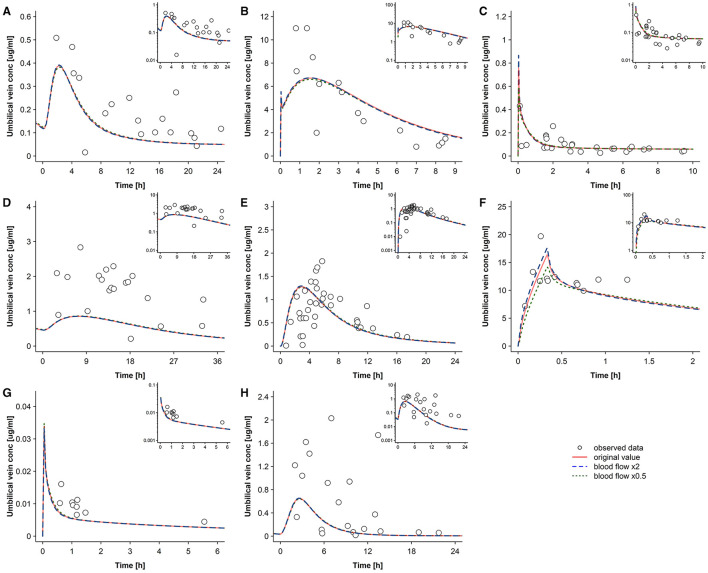
Sensitivity analysis for the maternal placental blood flow rate. Concentration-time profiles in the umbilical vein plasma for acyclovir **(A)**, cefuroxime **(B)**, diazepam **(C)**, dolutegravir **(D)**, emtricitabine **(E)**, metronidazole **(F)**, ondansetron **(G)**, and raltegravir **(H)** at delivery in the third trimester. Semi-log scaled figures are shown as inset plot in the top-right corner of each panel. The time refers to the time after drug administration. Circles represent individual observed data and the lines represents the predicted geomean concentrations with different blood flow rates to the maternal placenta. Observed data for acyclovir, cefuroxime, diazepam, dolutegravir, emtricitabine, metronidazole, ondansetron, and raltegravir were taken from Liu et al. (18), Leung et al. (39), Hirt et al. (40), Philipson and Stiernstedt (41), De Leeuw et al. (42), Elkomy et al. (43), Visser and Hund (44), Moore and McBride (45), Ridd et al. (46), and Mandelli et al. (47), respectively. conc, concentration.

**Figure 6:**
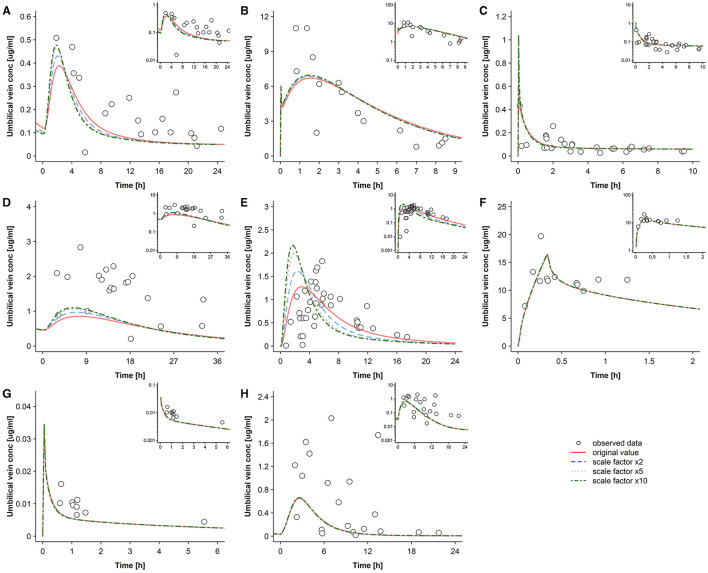
Sensitivity analysis for the transfer clearance across the apical trophoblast membrane where both influx and efflux clearance are equally varied. Concentration-time profiles in the umbilical vein plasma for acyclovir **(A)**, cefuroxime **(B)**, diazepam **(C)**, dolutegravir **(D)**, emtricitabine **(E)**, metronidazole **(F)**, ondansetron **(G)**, and raltegravir **(H)** at delivery in the third trimester. Semi-log scaled figures are shown as inset plot in the top-right corner of each panel. Circles represent individual observed data and the lines represents the predicted geomean concentrations with different transfer clearances across the apical trophoblast membrane (both influx and efflux transfer clearance are equally altered). Observed data for acyclovir, cefuroxime, diazepam, dolutegravir, emtricitabine, metronidazole, ondansetron, and raltegravir were taken from Liu et al. (18), Leung et al. (39), Hirt et al. (40), Philipson and Stiernstedt (41), De Leeuw et al. (42), Elkomy et al. (43), Visser and Hund (44), Moore and McBride (45), Ridd et al. (46), and Mandelli et al. (47), respectively. conc, concentration.

**Figure 7:**
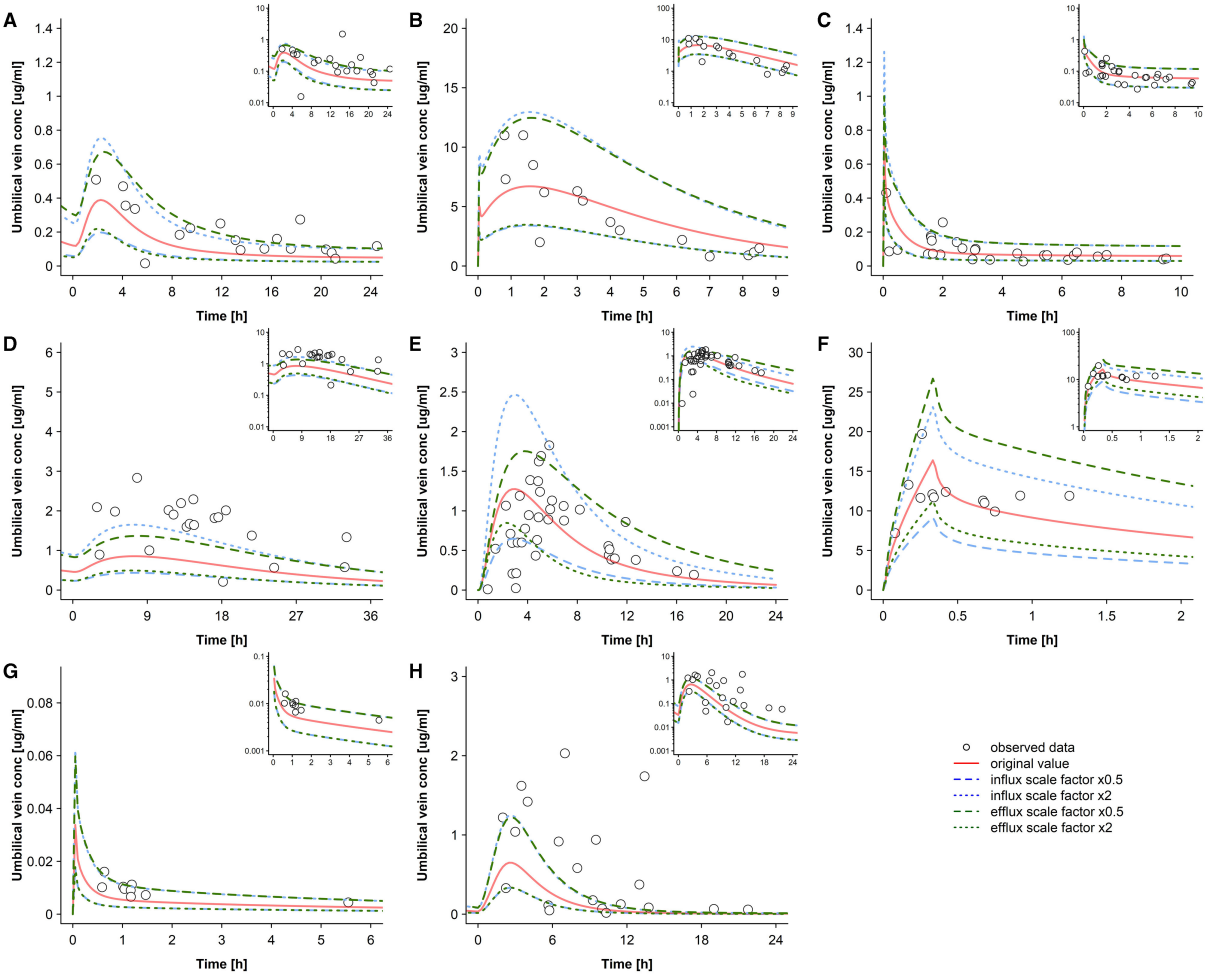
Sensitivity analysis for the transfer clearance across the apical trophoblast membrane where either influx or efflux clearance is varied. Concentration-time profiles in the umbilical vein plasma for acyclovir **(A)**, cefuroxime **(B)**, diazepam **(C)**, dolutegravir **(D)**, emtricitabine **(E)**, metronidazole **(F)**, ondansetron **(G)**, and raltegravir **(H)** at delivery in the third trimester. Semi-log scaled figures are shown as inset plot in the top-right corner of each panel. The time refers to the time after drug administration. Circles represent individual observed data and the lines represents the predicted geomean concentrations with different transfer clearances across the apical trophoblast membrane (either influx or efflux transfer clearance is altered). Observed data for acyclovir, cefuroxime, diazepam, dolutegravir, emtricitabine, metronidazole, ondansetron, and raltegravir were taken from Liu et al. (18), Leung et al. (39), Hirt et al. (40), Philipson and Stiernstedt (41), De Leeuw et al. (42), Elkomy et al. (43), Visser and Hund (44), Moore and McBride (45), Ridd et al. (46), and Mandelli et al. (47), respectively. conc, concentration.

**Figure 8:**
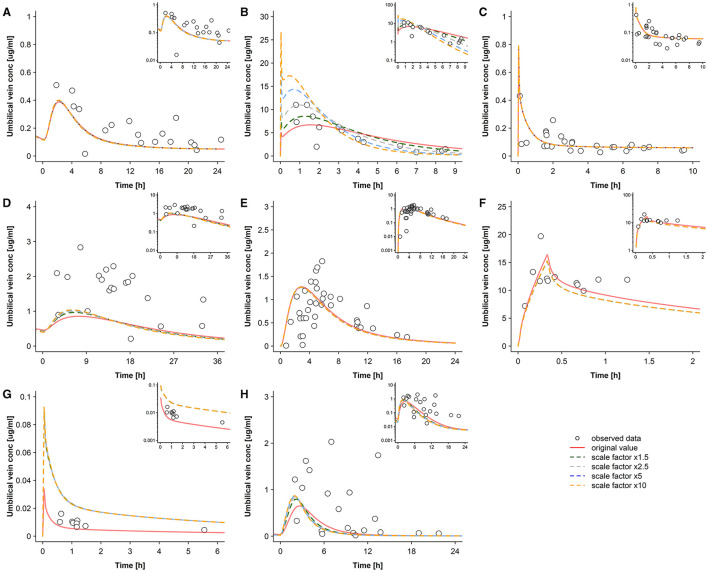
Sensitivity analysis for the transfer clearance across the basolateral trophoblast membrane. Concentration-time profiles in the umbilical vein plasma for acyclovir **(A)**, cefuroxime **(B)**, diazepam **(C)**, dolutegravir **(D)**, emtricitabine **(E)**, metronidazole **(F)**, ondansetron **(G)**, and raltegravir **(H)** at delivery in the third trimester. Semi-log scaled figures are shown as inset plot in the top-right corner of each panel. The time refers to the time after drug administration. Circles represent individual observed data and the lines represents the predicted geomean concentrations with different transfer clearances across the basolateral trophoblast membrane (both influx and efflux transfer clearance are equally altered). Observed data for acyclovir, cefuroxime, diazepam, dolutegravir, emtricitabine, metronidazole, ondansetron, and raltegravir were taken from Liu et al. (18), Leung et al. (39), Hirt et al. (40), Philipson and Stiernstedt (41), De Leeuw et al. (42), Elkomy et al. (43), Visser and Hund (44), Moore and McBride (45), Ridd et al. (46), and Mandelli et al. (47), respectively. conc, concentration.

Results of the sensitivity analysis are presented in Figures 5–**8**. Figure 5 shows the predicted concentration-time profiles in the umbilical vein when maternal placental blood flow rate was varied two-fold. Within this range, the maternal placental blood flow rate did not significantly affect the predicted umbilical vein concentrations except for metronidazole. For all drugs, the maximum difference in AUC_tlast_ between the original model and the model with altered blood flow rate did not exceed 1%, except for cefuroxime and metronidazole where the maximum difference was 2.9 and 5.7% when the blood flow rate was increased two-fold and reduced two-fold, respectively.

Figures 6, 7 present the concentration-time profiles in the umbilical vein that were predicted with different apical influx and efflux transfer clearance scaling factors (*f*_*in*_ and *f*_*out*_). As noted above, *f*_*in*_ and *f*_*out*_ modify the transfer clearance across the apical trophoblast membrane in the maternal-fetal and fetal-maternal direction, respectively. Changes in these parameters had only a negligible impact on maternal concentrations (data not shown). Figure 6 presents pharmacokinetic predictions when *f*_*in*_ and *f*_*out*_ are both varied equally, i.e., when the transfer clearance (*P*_*pls,cell*_ × *SA*_*villi*_) is similarly changed in both the influx and efflux direction. As was expected for orally administered drugs, except for raltegravir, higher values for *f*_*in*_ and *f*_*out*_ gave rise to greater peak concentrations (C_max_) and a lower time at which C_max_ is reached (t_max_). For intravenously administered drugs, variations in *f*_*in*_ and *f*_*out*_ only had a negligible effect on the predicted pharmacokinetic profiles.

Figure 7 shows predicted pharmacokinetic profiles when either *f*_*in*_ or *f*_*out*_ was changed, while the other one was kept unchanged at the original value of 1. It is important to note that these results are relative and will be different when the absolute transfer clearance (*P*_*pls,cell*_ × *SA*_*villi*_) is altered. As expected, these variations had a strong impact on the umbilical vein-to-maternal plasma concentration ratio. No consistent trend for under- or overestimation was found for the different drugs when apical influx or efflux transfer was altered. For some drugs (e.g., ondansetron and raltegravir), albeit not for all, a two-fold increase of the efflux transfer clearance showed results that were equivalent to a two-fold decrease in the influx transfer clearance.

The results for sensitivity analysis when varying the transfer clearance across the basolateral membrane are shown in Figure 8. These results were informative in that they revealed that the basolateral transfer clearance was also a sensitive parameter for some drugs, e.g., for cefuroxime and raltegravir.

## Discussion

Fetal therapeutics is rapidly becoming a reality both for drugs given to the mother and for gene and stem cell therapy delivered to the fetus. For drugs that are administered to the mother with the intent of treating the fetus, a further understanding of maternal to fetal drug transfer will be required. This study refined the ODE system of a previously published pregnancy PBPK model by implementing the fetal-specific unbound drug fraction in the model. Additionally, scaling factors were integrated in the model to account for asymmetrical drug transfer across the apical and basolateral membranes of the trophoblast. Using the refined model, maternal and fetal pharmacokinetics were predicted for eight different drugs. It was further investigated how different unbound drug fractions in the maternal and fetal circulatory system and different apical and basolateral influx/efflux transfer clearances impact the predicted drug concentrations in the umbilical vein.

In this study, apical transfer clearance was estimated using a previously proposed *in vitro-to-in vivo* extrapolation of a drug's passive diffusion clearance (30). This extrapolation approach appears attractive because *in vitro* permeability values required as input are often readily available in the literature. Although it was initially only proposed for drugs crossing the placenta exclusively *via* passive diffusion (30), this approach also yielded adequate results for ondansetron which is a substrate of P-glycoprotein [P-gp, also referred to as multidrug resistance protein 1 (MDR1)] (49), an efflux transporter expressed in the apical membrane of trophoblasts (50). On the other hand, for dolutegravir and raltegravir which are also P-gp substrates (51, 52), this approach substantially underestimated the clinical observations (Figure 4). Furthermore, for acyclovir, a substrate of various efflux transporters expressed in the placenta, such as the multidrug and toxin extrusion proteins (MATE) 1/2-K (53), it was expected that this approach would result in an overestimation of umbilical vein concentrations as efflux is not considered in the estimated apical transfer clearance value; yet, the presented results showed that an even higher placental transfer clearance would be required to adequately describe the data. Hence, the overall suitability of this approach for parameterizing placental transfer in PBPK models remains inconclusive. Importantly, a refined version of this approach has been proposed very recently (54) and merits further evaluation with additional drugs.

The predicted variability in maternal and, to a lesser extent, umbilical vein concentrations was generally underestimated (Figures 3, 4). To some extent, this was expected because the variability in anatomical and physiological parameters integrated in the PBPK models stems from observations in non-laboring women (29). While relatively little is known about changes in fetal physiological parameters during delivery, maternal physiological parameters, particularly those related to hemodynamics, show substantial variation during the peripartum period. For example, cardiac output is 13–31% higher in the first stage of labor compared to the pre-parturient level (55, 56) and can temporarily increase by ~20–60% during cesarean delivery, especially if uterotonics, e.g., oxytocin or carbetocin, are co-administered (57). This might give rise to temporary changes in various organ blood flows which could in turn contribute to increased variability in drug distribution and elimination. For drugs with a blood-flow limited distribution behavior across the placenta, such as metronidazole, a potential increase in maternal placental blood flow during labor can be expected to increase the distribution across the placenta leading to higher drug exposure in the fetus (further discussed below). Furthermore, elimination is relatively insensitive toward potential changes in liver blood flow for drugs that have a low to intermediate hepatic extraction ratio (all drugs investigated herein). For example, for diazepam, metronidazole, and ondansetron, a 30% increase in both hepatic arterial and portal vein blood flow increased clearance by only 0.3, 0.9, and 4.1%, respectively (data not shown). The clearance of high extraction drugs depends more on liver blood flow and might therefore be substantially increased during labor. While the effect of labor on cardiac output is relatively well-characterized, the effect on organ blood flows (e.g., for the placenta and liver) is unknown which complicates integrating these factors in a PBPK model.

Along a similar line, an overestimation of the maternal clearance of raltegravir and dolutegravir may have led to an underestimation of both maternal and fetal drug exposure. In these cases, increasing maternal plasma concentrations (e.g., by reducing UGT-mediated clearance or the unbound drug fraction) also improved predictions in the umbilical cord. This stresses again that maternal physiological changes need to be adequately captured in PBPK models since maternal and fetal pharmacokinetics are intimately connected.

The unbound drug fraction was estimated in this study from reported maternal and fetal albumin serum levels. Albumin does not cross the placenta (58, 59) and hence fetal albumin is exclusively of fetal origin. Fetal albumin is synthesized at a higher rate in the early third trimester vs. late third trimester (60) where the difference between fetal and maternal albumin concentrations diminishes. The findings of this study demonstrate that slight differences in maternal-fetal protein binding at term delivery generally have a rather small effect on the predicted umbilical cord concentrations (see Table 5). At quasi-equilibrium, it can be expected that the predicted concentration ratio between maternal and umbilical vein concentrations will approach the ratio of maternal to fetal fraction unbound.

When calculating the fetal unbound drug fraction according to Equation (1), it was assumed that the drug binds to one protein only (namely albumin) and that the number of adult and fetal protein binding sites as well as the drug's affinity to adult and fetal plasma proteins are the same. The assumption that albumin is the exclusive protein binding partner may contribute to an underestimation of the fetal unbound fraction of a drug with mixed binding to albumin or α1-acidic glycoprotein because the relative concentration difference between fetal and adult α1-acidic glycoprotein is considerably larger than that for albumin [α1-acidic glycoprotein: 0.21 g/L in the fetus at 38 weeks of gestation vs. 0.70 g/L in non-pregnant adults; albumin: 38.6 g/L in the fetus at 38 weeks of gestation vs. 46.4 g/L in non-pregnant adults (20, 29)]. Relatively little information could be found in the scientific literature to falsify (or verify) the assumption of equal number of protein binding sites and binding affinity of adult and fetal albumin. Investigating diazepam binding to albumin, Viani et al. (61) reported 0.83 and 1.02 number of albumin binding sites (expressed as moles of drug per mole of albumin) for fetal and adult serum, respectively, and an association constant of 1.36 × 10^−5^ and 1.00 × 10^−5^ M^−1^ to fetal and adult albumin, respectively. Calculation of the fetal unbound fraction from these values according to a previously described method (16, 19) yields a value of 0.024 for diazepam instead of 0.021 (Table 1) which is closer to the maternal value of 0.027 and would hence lead to a lower difference between predicted maternal and fetal plasma concentrations. Without further experimental data, though, it is difficult to evaluate the calculated fetal unbound fraction of the other investigated drugs. This highlights the need to further measure the fetal unbound fraction of diverse drugs in clinical samples and use these data to develop, train or validate methods for calculating the unbound fraction of a drug.

For drugs weakly or moderately bound to albumin (fraction unbound > ~30%), the differences in fetal/maternal protein binding can, under normal conditions, be expected to be rather low at term delivery because the difference between fetal and maternal albumin concentration diminishes toward term (20, 29). However, they may become more relevant at earlier stages of pregnancy. For example, in a paired analysis, Krauer et al. (62) observed that the fetal/maternal albumin concentration ratio was around 0.66 ± 0.30 (mean ± standard deviation) between 16 and 25 weeks of gestation and increased thereafter, reaching 1.20 ± 0.18 at >35 weeks of gestation. Additionally, differences in fetal/maternal fraction of unbound drug may be exaggerated in diseased states that have been observed to be associated with maternal or fetal hypoalbuminemia, such as preeclampsia and eclampsia (63) or severe hydrops fetalis (64). Finally, for drugs predominantly binding to α1-acidic glycoprotein, larger differences between maternal and fetal fraction of unbound drug may be expected as the observed fetal/maternal concentration ratio of α1-acidic glycoprotein rises only to 0.37 ± 0.23 (mean ± standard deviation) at term (62). This highlights that differential protein binding characteristics, although found to be generally only of minor importance in this study, might be relevant in various scenarios and should hence be structurally considered in PBPK models.

Interestingly, the observed pharmacokinetic profiles in the umbilical vein could not be captured for all drugs when placental transfer clearance, estimated from reported Caco-2 permeability (Table 2), was assumed to be equal in both maternal-fetal and fetal-maternal direction (Figure 4). For example, the umbilical vein concentrations of acyclovir, dolutegravir, and raltegravir were systematically underestimated and could not be improved when increasing the total blood flow to the placenta (Figure 5) or the total flux across the apical membrane (Figure 6). In fact, with the exception of metronidazole, the concentrations predicted in the umbilical vein were not sensitive to changes in the maternal placental blood flow, at least not within the tested range (Figure 5). These findings suggest that the distribution of these drugs across the placenta barrier is not limited by blood flow, but rather by the permeability through the trophoblasts' apical membrane at the fetal-maternal interface. This is an expected finding because for all drugs the product of the fraction of unbound drug (Table 1) and the apical transfer clearance rate (Table 2) is considerably lower than the mean placental blood flow of the mother (~0.75 L/min), except for metronidazole where the latter product amounts to 9.0 L/min which makes the transplacental distribution of metronidazole blood flow-limited. Hence, alterations in placental hemodynamics induced by labor and delivery might be of concern for this drug.

Although transfer clearance across the apical membrane was a sensitive parameter for orally administered drugs (except raltegravir), higher parameter values did not substantially improve the model performance (Figure 6). With equal apical transfer clearance in both influx and efflux direction, the ratio of predicted maternal to umbilical vein plasma concentrations at quasi-equilibrium was solely influenced by differential protein binding characteristics (Figure 4).

While the placental partition coefficients (Table 2) did influence intracellular concentrations in the trophoblasts, concentrations in the maternal and umbilical vein blood were not affected by this parameter (data not shown). For example, higher values for the maternal blood plasma-to-fetal intracellular partition coefficients led to higher intracellular drug concentrations in the trophoblast without significantly influencing maternal and umbilical vein plasma concentrations. This was expected because the values of the partition coefficient between maternal blood plasma and fetal intracellular space are similar to the values of the partition coefficient between fetal blood plasma and fetal intracellular space. Changes in umbilical vein concentrations will only be observed if the maternal blood plasma-to-fetal intracellular partition coefficient is changed while keeping the fetal blood plasma-to-fetal intracellular partition coefficient unchanged as has been shown previously (18, 48). The effect of such asymmetrical changes in placental partition coefficients is similar to alterations in the efflux transfer clearance; for example, as can be seen from Equation (14), a two-fold increase in *K*_*cell,pls*_ will yield the same results as reducing *f*_*out*_ by a factor of 0.5.

For several drugs (e.g., dolutegravir, ondansetron, and raltegravir), the clinical data could be better described when a higher influx-to-efflux transfer clearance ratio was applied in the models (Figure 7). Yet, it is difficult to draw general conclusions from these findings because they all relate to specific apical transfer clearance values (Table 2) which may be inaccurate as discussed above. For example, to improve the model predictions for dolutegravir, ondansetron and raltegravir, a higher influx-to-efflux ratio seemed to be necessary (Figure 7); however, since these drugs are P-gp substrates (49, 51, 52), a lower influx-to-efflux ratio would be biologically plausible. This might suggest that the applied *in vitro-to-in vivo* extrapolation approach underestimates the absolute apical transfer clearance for these drugs; in turn, a higher absolute apical transfer clearance could then accommodate a lower influx-to-efflux ratio. This hypothesis seems to be in line with findings from *in vitro* studies. When comparing various studies that quantified P-gp expression relative to that of the housekeeping gene GAPDH (glyceraldehyde-3-phosphate dehydrogenase), higher P-gp expression was found in Caco-2 cells (65–67) than in (syncytio)trophoblasts (68, 69). These expression data corroborate the hypothesis that, compared to Caco-2 cells, a weaker effect of P-gp mediated efflux can be expected for the placenta barrier. As can be seen from Equation (14), it is unfortunately not possible to use these PBPK models for estimation of both the apical transfer clearance and the factor modulating efflux clearance (*f*_*out*_) because of non-identifiability issues.

Even if the transfer clearance across the apical membrane is accurately parameterized, the transfer clearance across the basolateral membrane of the trophoblast may also play an important role as was found, e.g., for cefuroxime and ondansetron (Figure 8). In the presented PBPK models, the basolateral transfer clearance was estimated as product of the drug's organ permeability (2.73 × 10^−6^ and 1.69 × 10^−2^ cm/min for cefuroxime and ondansetron, respectively) and the surface area between the trophoblasts' intracellular and interstitial space (on average ~56,700 dm^2^). For cefuroxime, but not for ondansetron, the resulting basolateral transfer clearance was lower than the apical transfer clearance (0.015 vs. 0.20 L/min for cefuroxime and 96 vs. 3.11 L/min for ondansetron). These findings illustrate that an adequate parameterization of placental drug transfer should consider both apical and basolateral transfer clearance rates.

In addition to the model parameters investigated herein (placental blood flow, apical and basolateral influx and efflux clearance rates and differential protein binding in mother and fetus), the degree of a drug's ionization could also affect placental transfer as only the non-ionized drug fraction can cross the trophoblast membrane. The pH of the fetal blood is ~0.1 log unit lower than that of the maternal blood. Although this is generally of less concern under normal conditions, the pH difference may be exaggerated in the case of fetal asphyxia, or in situations of severe maternal hemorrhage and coagulopathy requiring blood transfusions (70). In the case of weakly basic drugs, a lower pH value of the fetal blood is associated with a higher fraction of the ionized form of the drug leading to ion trapping and higher drug concentrations in the fetal blood as has been observed e.g., for bupivacaine (71) and lidocaine (72). Among the drugs investigated herein, only ondansetron is weakly basic with a pKa of 7.80 (73) which might have partly contributed to the underestimation of ondansetron concentrations in the umbilical vein.

As stated above, it is difficult to accurately identify mechanisms of the misfit between predicted and observed pharmacokinetics because multiple factors can affect the predictions in a similar fashion. For example, ondansetron pharmacokinetics could be better described by either a higher fetal fraction unbound; a higher ratio of influx-to-efflux transfer clearance across the apical membrane; a higher transfer clearance across the basolateral membrane; by potentially accounting for the different pH value in fetal blood; or by a combination of all these factors. This indicates that further clinical data of well-characterized drugs are required to systematically inform placental blood flow rates, passive and active transfer processes as well the effect of differential protein binding and pH values between maternal and fetal blood.

Another limitation of the presented maternal-fetal PBPK models is the lack of a mechanistic integration of drug transporters in the placenta. On a physiological level, the differences in influx/efflux diffusion clearances might be attributed to the presence of drug transporters in the placenta. The presented findings highlight the fact that an adequate parametrization of transporter activities in the apical and basolateral membrane of the trophoblasts is crucial for predicting fetal drug exposure. Currently, the integration of placental transporters in PBPK models is hampered by the scarce information on transporter abundance in the (syncytio)trophoblasts.

Additionally, placental metabolism has not been accounted for in this study. Yet, the expression or activity of numerous drug-metabolizing enzymes has been found to be absent in the human term placenta (74, 75). In fact, the enzymes involved in the metabolism of the drugs investigated herein—except for acyclovir and emtricitabine—are either not expressed or not functionally active in the term placenta. To the best of our knowledge, the expression of aldehyde oxidase, which is responsible for metabolism of acyclovir [~10% of the dose in non-pregnant adults (15)], has not yet been studied in the human term placenta, while the enzyme involved in metabolism of emtricitabine [~29% of the dose in non-pregnant adults (15)] is not identified. Therefore, it appears unlikely that placental transfer of the investigated drugs could have been influenced by placental metabolism. Still, for other drugs, especially those with a high extraction ratio, placental metabolism, if present *in vivo*, should be accounted for in the model as this would potentially decrease the net flux of drug across the placenta.

Finally, this study was limited to eight drugs. It is evident that further models and additional clinical data from both mother and fetus are needed to further advance our understanding of placental drug transfer. While pregnant women have historically been excluded from clinical trials, the lack of drug studies in pregnant women has been recognized as a major health issue (76). There seems to be a slow paradigm shift arguing in favor of the inclusion of pregnant women in clinical research (77–79) which is also, at least to some extent, reflected by recent guidance documents from the US Food and Drug Administration (FDA) (7). Hence, it can be expected that more clinical data in pregnant women will be generated within the next years. Analyzing these data with modeling and simulation techniques will help to interpret these data by mathematical abstraction and thus generate further insights in maternal-fetal pharmacology.

In conclusion, our current understanding of drug transfer kinetics across the placenta is only rudimentary. The findings indicate that, in the late third trimester, differential protein binding characteristics in the maternal and fetal system give rise to only minor differences in maternal-fetal exposure to albumin-bound drugs, especially if protein binding is low or moderate. Differences in placental influx and efflux clearance, however, were found to be highly relevant stressing the importance of drug transporters in the placenta. Hence, further clinical studies are required to better disentangle and quantify both passive and active transfer processes across the apical and basolateral membrane of the trophoblast. This updated PBPK model structure is freely shared on OSP GitHub (https://github.com/Open-Systems-Pharmacology) for further applications and/or refinements that were beyond the scope of this study. Ultimately, once the confidence in maternal-fetal PBPK models has been established, these models might be, among other approaches, a powerful tool to support informed decision making for a safe and efficient pharmacotherapy targeting the mother and/or fetus.

## Data Availability Statement

The original contributions presented in the study are publicly available. This data can be found here: GitHub (https://github.com/Open-Systems-Pharmacology).

## Author Contributions

AD designed the research. XL, GB, and AD wrote the manuscript. XL and AD performed the research. XL, DG, JA, NR, HA, JM, KP, GB, and AD analyzed the data. All authors contributed to the article and approved the submitted version.

## Conflict of Interest

AD is an employee of Bayer AG and uses Open Systems Pharmacology software, tools, and models in his professional role. The remaining authors declare that the research was conducted in the absence of any commercial or financial relationships that could be construed as a potential conflict of interest.

## Publisher's Note

All claims expressed in this article are solely those of the authors and do not necessarily represent those of their affiliated organizations, or those of the publisher, the editors and the reviewers. Any product that may be evaluated in this article, or claim that may be made by its manufacturer, is not guaranteed or endorsed by the publisher.
